# In Vitro Fermentation Characteristics of Fungal Polysaccharides Derived from *Wolfiporia cocos* and Their Effect on Human Fecal Microbiota

**DOI:** 10.3390/foods12214014

**Published:** 2023-11-02

**Authors:** Ka Lee Ma, Nelson Kei, Fan Yang, Susana Lauw, Po Lam Chan, Lei Chen, Peter Chi Keung Cheung

**Affiliations:** 1 Food and Nutritional Sciences Program, School of Life Sciences, The Chinese University of Hong Kong, Hong Kong SAR, China; kellyma@link.cuhk.edu.hk (K.L.M.); nelsonkei@link.cuhk.edu.hk (N.K.); susana.lauw@link.cuhk.edu.hk (S.L.); polamchan@cuhk.edu.hk (P.L.C.); 2 Biochemistry Program, School of Life Sciences, The Chinese University of Hong Kong, Hong Kong SAR, China; 1155134641@link.cuhk.edu.hk; 3 Key Laboratory of Carbohydrate Chemistry and Biotechnology, Ministry of Education, School of Biotechnology, Jiangnan University, Wuxi 214122, China; leichen@jiangnan.edu.cn; 4 National Engineering Research Center of Cereal Fermentation and Food Biomanufacturing, Jiangnan University, Wuxi 214122, China

**Keywords:** gut microflora composition, in vitro fermentability, beta glucan, Fu Ling, prebiotics

## Abstract

Gut microbiota has been described as a new ‘organ’ that interferes with host physiology by its metabolites produced from the utilization and biotransformation of undigested food components. Fu Ling (FL), the sclerotia of fungi *Wolfiporia cocos*, contains β-glucan, which is a known natural polysaccharide with strong medicinal efficacy. This study endeavors to evaluate the fermentability of FL and polysaccharides extracted from its sclerotia. An in vitro fermentation of structurally characterized FL and its β-glucan by human fecal microbiota was conducted. Total bacterial count, pH change, short-chain fatty acid profile and microbiota profile were assessed post-fermentation. FL containing over 70% of β-(1 → 3) and (1 → 6)-glucans with a low degree of branching of 0.24 could enhance acetic acid (a major microbial metabolite) production. Both FL and its extracted β-glucan had similar modulation on microbial composition. They enriched *Phascolarctobacterium faecium*, *Bacteroides dorei* and *Parabacteroides distasonis*, all of which are shown to possess anti-inflammatory effects. FL polysaccharide can be utilized as a natural whole food for its potential health benefits to human gut bacteria.

## 1. Introduction

*Wolfiporia cocos*, formerly known as *Poria cocos*, is an edible medicinal fungus which belongs to the phylum Basidiomycota. Distinct from most edible mushroom-forming fungi, *W. cocos* forms large sclerotium on the roots of pine trees or on pine woods under agricultural cultivations. Its sclerotium is referred to as Fu Ling (FL), Hoelen, Indian bread, Poria or Tuckahoe, depending on the area. It is widely used in traditional medicine in Asian countries, including China, Japan, Korea and India, and North America, due to its remarkable immunomodulating, anti-oxidative, anti-inflammatory, anti-tumor, anti-hepatitis and anti-diabetic activities. It contains mainly polysaccharides and small amounts of triterpenoids, fatty acids, sterols and enzymes, while the majority of its natural polysaccharides are β-glucans [1,2,3]. Although FL is conventionally utilized in medicinal decoctions in its crude form, it has seldom been included in studies. Plenty emphasize its components, particularly β-glucan and triterpenoids, instead.

β-glucans are polysaccharides consisting of a chain of linear or branching monosaccharides linked via β-glycosidic bonds. The pharmacological effects and bioavailability of β-glucans vary greatly depending on their origin. Cereals, fungi, and yeast are rich sources of natural β-glucan found in our diet. However, their content or extraction yields vary widely from 5 to 90%. Oats and barley have a low level of about 5%. Yeast contains a considerable amount, but the extraction yields only 5–7% due to their rigid cell wall. Members of fungal mushrooms contain about 3 to 46% of those [4]. Sclerotia of fungi, as a mass of mycelia, have a remarkably high level of β-glucans of approximately 80% to over 90% [5,6,7]. Cereal β-glucans from oat and barley are water-soluble and mix-linked via β-(1 → 3)-(1 → 4) linkages with no branching [8,9]. Fungal (including mushroom) and yeast β-glucans share the features of the β-(1 → 3) backbone and β-(1 → 6) side chain. However, fungal β-glucans have short branches, whereas yeast β-glucans have additional β-(1 → 3) regions. They may be soluble or insoluble in water due to other factors [9,10]. Significant effort has been devoted to the structure and function relationship in β-glucans in the past decades. Structural differences in backbone, side chain, degree, length and position of branching, spatial conformation and monosaccharide compositions determine their water solubility and binding affinity to receptors, and thus their biological properties [9,10,11]. Some studies found the modification of FL β-glucans necessary to exhibit the mentioned benefits for the same reason. Many studies and reviews have reported their close causality, while unexplained exceptions still exist.

Although FL β-glucans have poor water solubility prior to chemical modification, their fermentability is still controversial. Yang et al. believe that they are poorly fermented because bacteria can hardly adhere to their flat surface [12]. Wong et al. found them to be fermentable, with over 50% organic matter disappearance, and found that they produced a considerable amount of short-chain fatty acids (SCFAs) after 24 h in vitro fermentation by human fecal microbiota [13]. Regardless of a β-glucan’s solubility in water, the numerous hydroxyl groups readily form hydrogen bonds with water molecules, giving it excellent water-holding properties and strong hydrophilicity [8]. The considerable swelling of insoluble β-glucan solution delays gastric emptying and prolongs intestinal transit, which may favor colonic fermentation. Some bacteria encode carbohydrate-active enzymes (CAZymes) with specific carbohydrate binding modules (CBM) that enhance their binding affinity to substrates, including those insoluble in water. For instance, β(1 → 3)-glucanases of *Chitinophaga pinensis* target and recognize water-insoluble chitin and β-glucans found in fungal cell walls [8,14].

The gut microbiota, known as intestinal microbiota, gut flora or intestinal flora, refers to a complex community of about 100 trillion mainly bacteria colonized in the host’s gastrointestinal tract. They belong to over 1000 species and carry 2,000,000 genes, which is 100 times that of human genes. The gut microbiota influences host digestion, the brain, emotions, immune system, cardiovascular health and hormone secretion. Significant alteration in gut microbiota composition is often observed in diseased patients. Such dysbiosis is even described as a potential microbial biomarker for precision medicine [15,16,17]. Indigestible food remains in the colon could be a potential substrate for gut microbes. They utilize the food substances to release energy for their growth and unwanted modified metabolites. Gut microbiota convert ginsenosides in ginsengs into compound K, a much more potent anti-tumor, anti-inflammatory, anti-diabetic, anti-allergic and neuroprotective compound [18]. The fermentability of human gut microbiota is a potential mechanism to activate food compounds and release powerful pharmacological effects. For these reasons, the present study aims to evaluate the change in bacterial growth, pH, SCFAs and microbial composition brought about by FL and its main polysaccharide, β-glucan, after human gut microbiota fermentation.

## 2. Materials and Methods

### 2.1. Identification and Authentication of Fu Ling

Fresh and unprocessed sclerotia of *Wolfiporia cocos* (Fu Ling) from Yunnan Province in China were purchased from a local Chinese medicine store. Sclerotia were washed to remove dust and soil, followed by air-drying overnight. Fresh samples of inner mycelial mass were obtained and cryogenically grinded with mortar and pestle. DNA was extracted using a lysis buffer containing 2% cetyltrimethylammonium bromide, 100 mM Tris-HCl, 1.4 M NaCl, 20 mM EDTA and 0.2% β-mercaptoethanol, and UltraPure Phenol:Chloroform:Isoamyl Alcohol (25:24:1, *v*/*v*) (15593031, Invitrogen, Waltham, MA, USA). Extracted genomic DNA was concentrated and cleaned up using AMPure XP Bead-Based Reagent (A63881, Beckman Coulter, Indianapolis, IN, USA), amplified for the ITS region with ITS 1 and ITS 4 oligonucleotide primers (Table 1) using KAPA HiFi HotStart ReadyMix (KK2602, Roche Molecular Systems, Inc., Basel, Switzerland) and sequenced by Sanger sequencing. The sequence obtained was uploaded to the Basic Local Alignment Search Tool (BLAST) for species annotation using Nucleotide BLAST [19].

### 2.2. Preparation of Materials

#### 2.2.1. Preparation of Fu Ling

Washed and dried sclerotia were peeled to remove the brown outer skin and pale pink inner skin. Any samples mixed with wood or invaded by insects were excluded. The remaining healthy samples were further dried and pulverized with a cyclotech mill (Tecator, Höganäs, Sweden). Fine powder passing through a 0.5 mm sieve was collected as crude Fu Ling powder, which hereafter is referred to as FL. FL was stored in an airtight desiccator at room temperature prior to further usage. Some FL powder was used for alkaline extraction to obtain its β-glucan.

#### 2.2.2. Extraction of FL β-Glucan

FL was defatted in 1:10 *w/w* of ethyl acetate for 2 h and acetone for another 2 h. The dried and defatted powder was soaked in 1:20 *w/w* of 1 M sodium hydroxide for at least 16 h at room temperature with continuous agitation. The supernatant of the alkali-soluble fraction was neutralized with hydrochloric acid to yield jelly-like precipitates, which were then washed 5 times using Milli-Q water [20]. The washed precipitates were dialyzed (Spectra/Por molecularporous membrane tubing of MWCO 6–8 kDa, d = 32 mm) against Milli-Q water until the conductivity of the water-soaking dialysis membranes was below 10 µS/cm, as measured using a conductivity/TDS meter. The dialyzed solution was lyophilized to obtain large fragments of alkali-soluble β-glucan. They were grinded into fine powder in liquid nitrogen and lyophilized again to remove moisture condensed under low temperature.

### 2.3. Monosaccharide Composition Analysis

#### 2.3.1. Hydrolysis and Alditol Acetate Conversion

FL and FL β-glucan underwent modified Saeman hydrolysis, consisting of hydrolysis in 12 M and 2 M sulphuric acid with stirring [21]. Allose was added to the acid hydrolysates as an internal standard before reduction and acetylation [22]. Reduction was conducted with the aid of freshly prepared sodium borohydride (reducing agent) and octan-1-ol (anti-foaming agent) under an alkaline condition created by 12 M ammonia. Acetic anhydride (acetate donor) and 1-methylimidazole (catalyst) were involved in the acetylation process. The resultant alditol acetates were extracted in dichloromethane (DCM) with 3 washings using Milli-Q water. The extracts were stored in GC vials at −20 °C after filtering through a 0.45 µm membrane.

#### 2.3.2. Gas Chromatography-Flame Ionization Detection (GC-FID) Analysis

Various sugar derivatives were separated by an HP 6890 series GC system equipped with an Agilent 122-2212 DB-225 capillary column (15 m × 0.25 mm; i.d. 0.25 µm film, Agilent Technologies, Santa Clara, CA, USA) and a flame ionization detector. Two µL of each sample were injected with a split ratio of 20:1. The oven temperature was programmed to an initial temperature of 170 °C, followed by a temperature rise of 2 °C/min to 220 °C, with a final hold of 15 min. The injector and detector were set as 250 °C, with helium as the carrier gas flowing at 1 mL/min. Sugar derivatives were identified with reference to a standard mix of arabinose, focuse, galatose, galacosamine, glucosamine, glucose, mannose, rhamnose, ribose and xylose.

### 2.4. β-Glucan Content

The β-glucan content of FL and the extracted β-glucan were determined using the Megazyme β-Glucan Assay Kit (Yeast and Mushroom) (Cat no.: K-YBGL). In brief, the total glucan was measured by dissolving β-(1 → 3)-glucans, β-(1 → 3), (1 → 6)-glucans and α-glucans in ice cold 12M sulphuric acid, followed by hydrolyzation to glucose with the aid of exo-1,3-β-glucanase and a β-glucosidase enzyme mix. The free glucose and released glucose were determined using a GOPOD reagent, which induced a color change to be measured by an absorbance microplate reader (BMG CLARIOstar Microplate Reader, BMG Labtech, Ortenberg, Germany). The α-glucan content was measured by degrading α-glucan with amyloglucosidase, invertase and trehalase. The free glucose and released glucose were again measured using a GOPOD reagent and absorbance microplate reader. The β-glucan content was calculated by subtraction of the α-glucan content from the total glucan content.

### 2.5. Glycosidic Linkage Analysis

#### 2.5.1. Partially Methylated Alditol Acetate (PMAA) Conversion

The NaOH/CH_3_I methylation method was employed to assess the various linkages present, which involved a series of chemical reactions as briefly described below. First, unlinked carbohydrate hydroxyls were converted to alkoxides with sodium hydroxide/dimethyl sulfoxide slurry, methylated with iodomethane and extracted with DCM. Secondly, the samples were hydrolyzed to monosaccharides using 2 M trifluoroacetic acid at 100 °C in an oven overnight. They were washed with methanol 3 times and dried under nitrogen flow in a Reacti-Vap^TM^ Evaporators system (Thermo Fisher Scientific Inc, Waltham, MA, USA). Thirdly, monosaccharides were reduced to alditols with 100 mg/mL sodium borohydride dissolved in 2 M ammonia solution. Glacial acetic acid was utilized to stop the reduction. Lastly, free hydroxyls were acetylated with 1-methylimidazole and acetic anhydride. Water was added to stop the acetylation. The resultant PMAAs were extracted in DCM and dehydrated with anhydrous sodium sulfate before being filtered through a 0.45 µm membrane into GC vials [23].

#### 2.5.2. Gas Chromatography-Mass Spectrometry (GC-MS) Analysis

Various PMAAs were separated by an HP 6890 series GC system equipped with an Agilent J&W HP-5ms GC column (30 m × 0.25 mm; i.d. 0.25 µm film). The oven temperature was programmed to an initial temperature of 50 °C with a hold of 2 min, followed by a rapid temperature rise of 30 °C/min to 150 °C and another temperature rise of 3 °C/min to 220 °C, with a final hold of 10 min at 300 °C. The injector and detector were set as 250 °C, with helium as the carrier gas flowing at 1 mL/min. Mass spectrometry acquisition was programmed to scan from 50–500 m/z in electron-impact mode. Linkages were identified with reference to the Complex Carbohydrate Research Centre Spectral Database for PMAAs (https://glygen.ccrc.uga.edu/ccrc/specdb/ms/pmaa/pframe.html) accessed on 26 September 2022.

### 2.6. Human Fecal Microbiota Collection

Fresh human feces were collected separately from three healthy donors, one female and two males, aged 22 to 26, who had no history of bowel disorders and usage of antibiotics for at least 3 months prior to fecal collection. The protocols were approved by The Joint Chinese University of Hong Kong—New Territories East Cluster Clinical Research Ethics Committee (Ref. No: 2019.165). The donors were allowed to follow their own daily diet to maintain a normal gut workload. The feces collected were preserved in 1:1 *v/v* of sterile stabilizing buffer consisting of 0.9% sodium chloride and 30% glycerol to maintain bacteria viability and 0.1% cysteine hydrochloride to maintain anaerobicity. The fecal samples were transported at −20 °C and stored at −80 °C until fermentation [24].

### 2.7. In Vitro Fermentation

Just prior to fermentation, one volume of stabilizing buffer was added to each fecal sample to make a 1:2 *v*/*v* fecal solution. They were then pooled, mixed thoroughly and filtered through several layers of sterile cheesecloth to obtain a fecal slurry.

Fermentations were performed in modified medium for colon bacteria (mMCB), which served as a basal medium to support the growth of human colon bacteria without a carbon source [24,25]. mMCB contained 6.5 g of bacteriological peptone, 5 g of soy peptone, 2.5 g of tryptone, 3 g of yeast extract, 2 g of KCl, 0.2 g of NaHCO_3_, 4.5 g of NaCl, 0.5 g of MgSO_4_ ·7H_2_O, 0.45 g of CaCl_2_ ·2H_2_O, 0.2 g of MnSO_4_ ·H_2_O, 0.005 g of FeSO_4_ ·7H_2_O, 0.005 g of ZnSO_4_ ·7H_2_O, 0.4 g of L-cysteine-HCl, 0.005 g of hemin, 0.005 g of menadione, 0.5 mL of H_3_PO_4_ and 2 mL of Tween 80 per liter. FL and FL β-glucan were added as the sole carbon source. No carbon source was added in the negative control. For each fermentation, 1% *w/v* of the interested carbon source was mixed with 10% fecal bacterial inoculum and mMCB. Fermentation was conducted in triplicates in an airtight anaerobic jar containing Oxoid^TM^ AnaeroGen^TM^ (AN0025A, Thermo Fisher Scientific Inc., Waltham, MA, USA) under 37 °C for 24 h. Aliquots of fermentation samples were collected for bacterial plate counting, pH measurement, short-chain fatty acid profiling and microbiota profiling.

### 2.8. Bacterial Growth

Bacterial growth was determined by the standard plate count method using BD Difco^TM^ Plate Count Agar (Becton, Dickinson and Company, Franklin Lakes, NJ, USA) in duplicate immediately after fermentation. In short, fermentation aliquots were serially diluted with sterile PBS. In each petri dish, 100 µL of sample was inoculated using the pour plate method. They were incubated in an airtight anaerobic box containing Oxoid^TM^ AnaeroGen^TM^ (Thermo Fisher Scientific Inc, Waltham, MA, USA) under 37 °C for 48 h. The growth of bacteria was expressed as CFU/mL fermentation mixture.

### 2.9. pH and Short-Chain Fatty Acids (SCFAs) Production

pH values before and after fermentation were measured with a pH meter immediately after collection. SCFAs were analyzed using GC-FID [13]. A total of 700 µL of frozen aliquots of fermentation medium were thawed and acidified with 175 µL of 25% meta-phosphoric acid, 5 µL of internal standard and 4-methyl valeric acid (100 mg/mL of 25% meta-phosphoric acid). The mixtures were incubated at room temperature for 30 min with continuous agitation. SCFAs were extracted in 1 mL of diethyl ether twice and dehydrated with anhydrous sodium sulfate. A standard mixture of acetic acid, propionic acid, butyric acid and valeric acid was prepared following the sample extraction procedures. SCFAs samples were stored at −20 °C after filtering through a 0.45 µm membrane into GC vials.

SCFAs were quantified by an HP 6890 series GC system equipped with an Quadrex 007-FFAP capillary column (30 m × 0.25 mm; i.d. 0.25 µm film) connected to a flame ionization detector. Five µL of each sample was injected with a split ratio of 20:1. The oven temperature was programmed to an initial temperature of 80 °C with a hold of 3 min, followed by a temperature rise of 1 °C/min to 100 °C with a hold for 5 min and another temperature rise of 10 °C/min to 200 °C with a final hold for 4 min. The injector and detector were set as 220 °C, with nitrogen as the carrier gas flowing at 1.5 mL/min. The change of SCFAs after the 24 h fermentation was expressed as µmol/L fermentation medium.

### 2.10. Microbial Composition

Fermentation aliquots were centrifuged to collect pellets. DNA was extracted using QIAamp Fast DNA Stool Mini Kit (51604, Qiagen, Hilden, Germany) and amplified for 16S (V1-V9) regions using tailed primers (Table 2) with the aid of KAPA HiFi HotStart ReadyMix (KK2602, Roche Molecular Systems, Inc., Basel, Switzerland). Purified amplicons were barcoded, end-repaired, ligated with an adapter and sequenced on Oxford Nanopore Technologies MinION flow cell according to the manufacturer’s instruction on ligation sequencing amplicons with PCR barcoding (SQK-LSK109, EXP-PBC096, Oxford Nanopore Technologies, Oxford, United Kingdom; E7180S, New England Biolabs, Ipswich, MA, USA). Data were basecalled and demultiplexed with MinKNOW and analyzed with EPI2ME 16S workflow developed by the same company.

The Shannon Index and Simpson Index were employed to calculate the α-diversity of samples. These indices summarize information on species richness and species abundance. Their formulas are shown below.

(1) $$Shannon\;Index=-\sum_{i=1}^{s}p_{i}\ln p_{i}$$

(2) $$Simpson\;Index=\frac{1}{\sum_{i=1}^{s}{p_{i}}^{2}}$$

where *p* is the proportion (n/N) of individuals of one species found (*n*) divided by the total number of individuals found (N), ln is the natural log, Σ is sum of the calculation and *s* is the number of species.

### 2.11. Statistical Analysis

The data were expressed as the mean ± SD of the three experiments and statistically analyzed using a two-tailed Student t-test for β-glucan content in BG and FL and a one-way ANOVA, followed by post hoc Tukey’s multiple comparisons test in post-fermentation assessments, at the significance level of *p* < 0.05, using GraphPad Prism 9.4 (GraphPad Software Inc., La Jolla, CA, USA).

## 3. Results

### 3.1. DNA Authentication

The BLAST results confirmed the identity of *Wolfiporia cocos*. The forward strand gave *W. cocos* at 87.17% sequence identity with a low E value, yet a low query coverage. The reverse strand gave *W. cocos* at 95.25% sequence identity with a 0.0 E value and a high query coverage of 83%.

### 3.2. Structural Characterization of Fu Ling (FL) and β-Glucan

**Figure 1:**
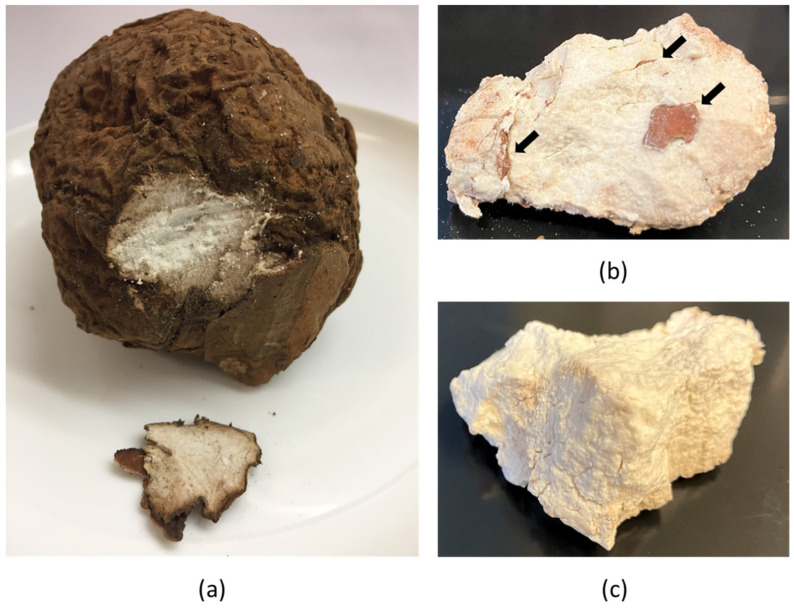
(**a**) Fresh Fu Ling sample with peel; (**b**) wood pieces (black arrows) embedded inside mycelial mass; (**c**) sample collected and used in the study.

The fresh and unprocessed FL was a high-density, round-shaped and stone-like sclerotium. The samples found with wood substrate embedded inside as mycelial growth or insect invasion were excluded. The remaining FL collected were white to pale yellow in color (Figure 1).

The polysaccharide of FL contained 73.1% total glucan, of which 99.9% was composed of a β-(1,3)-linked glucose backbone and very few β-(1,6)-linked short side chains without monosaccharides other than glucose. It had a degree of branching of 0.09. Thus, the FL polysaccharide is referred to β-glucan (BG) in this study. Before alkali extraction, FL itself contained a high percentage of total glucan of 76.5% as well. Similarly, 99.3% were β-(1 → 3), (1 → 6)-glucans with trace amounts of arabinose (5.4%) and mannose (1.6%) other than glucose (93.0%). It had a slightly higher degree of branching of 0.24. FL and its BG had a highly resembling structure and monosaccharide composition (Table 3, Table 4 and Table 5).

### 3.3. Post-Fermentation Assessments

**Figure 2:**
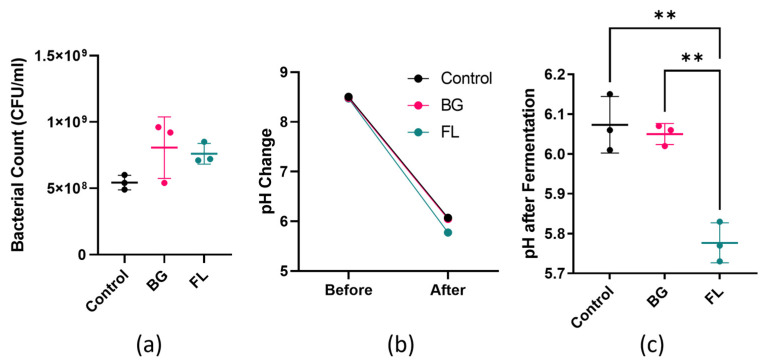
(**a**) Bacterial count (in duplicate) on plate count agar, expressed as CFU/mL; (**b**) pH change before and after 24 h fermentation; (**c**) pH comparison after fermentation. Results are expressed by mean ± SD, analyzed using one-way ANOVA, followed by post hoc Tukey’s multiple comparisons test. ** represents *p* < 0.01.

An increasing trend in the bacterial count in FL was observed when compared to the negative control without an added carbon source. The pH values of all groups dropped after fermentation, but that of FL was significantly lower than the control and BG, indicating the production of acidic metabolites (Figure 2).

**Figure 3:**
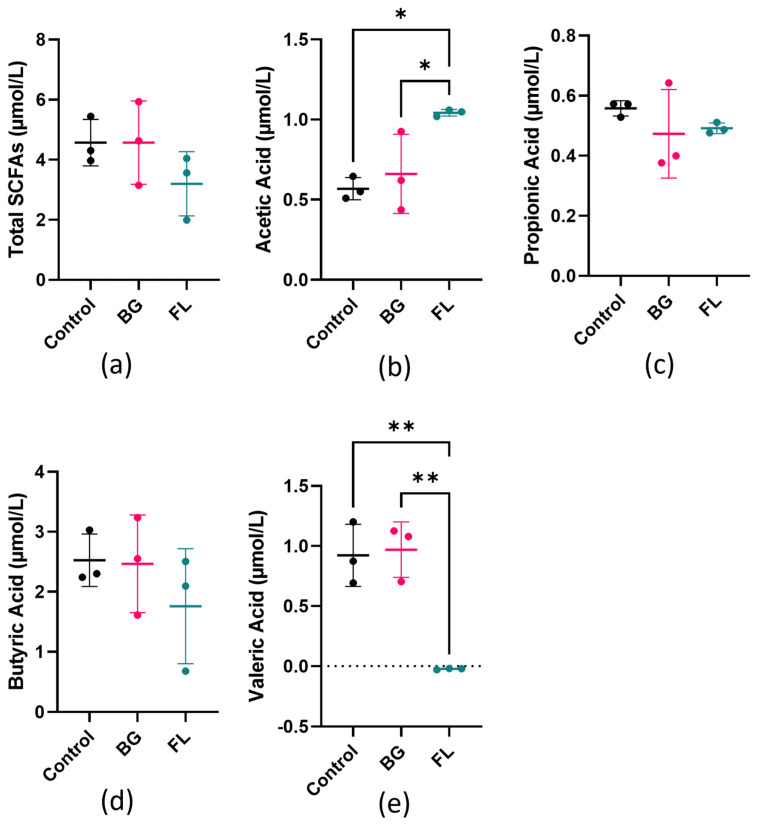
(**a**) Sum of changes in acetic acid, propionic acid, butyric acid and valeric acid, expressed as µmol/L fermentation matrix; (**b**–**e**) acetic acid, propionic acid, butyric acid and valeric acid changes before and after 24-h fermentation, expressed as µmol/L fermentation matrix, respectively. Results are expressed by mean ± SD, analyzed using one-way ANOVA, followed by post hoc Tukey’s multiple comparisons test. * represents *p* < 0.05; ** represents *p* < 0.01.

The change in the short-chain fatty acids (SCFAs) profile was examined. The total SCFA production did not differ much in all groups, while their individual major SCFAs differed. FL doubled acetic acid and slightly reduced propionic acid production compared to the control. Butyric acid production was similar across all groups. No net change was observed in the valeric acid amount in FL when the medium supported some production, implying an expenditure of valeric acid (Figure 3).

### 3.4. Modulation of Human Fecal Microbiota

**Figure 4:**
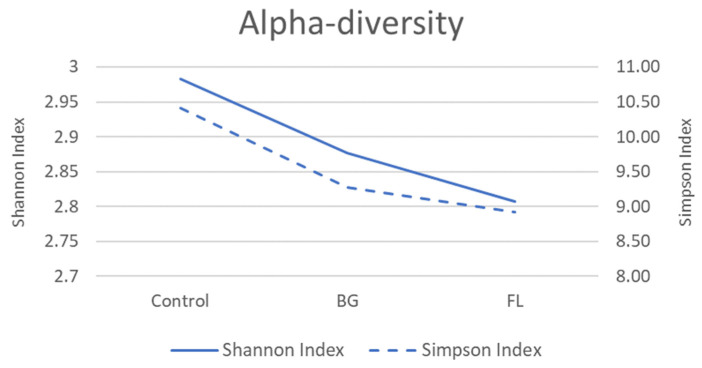
Line plot of α-diversity calculated using Shannon Index and Simpson Index.

**Figure 5:**
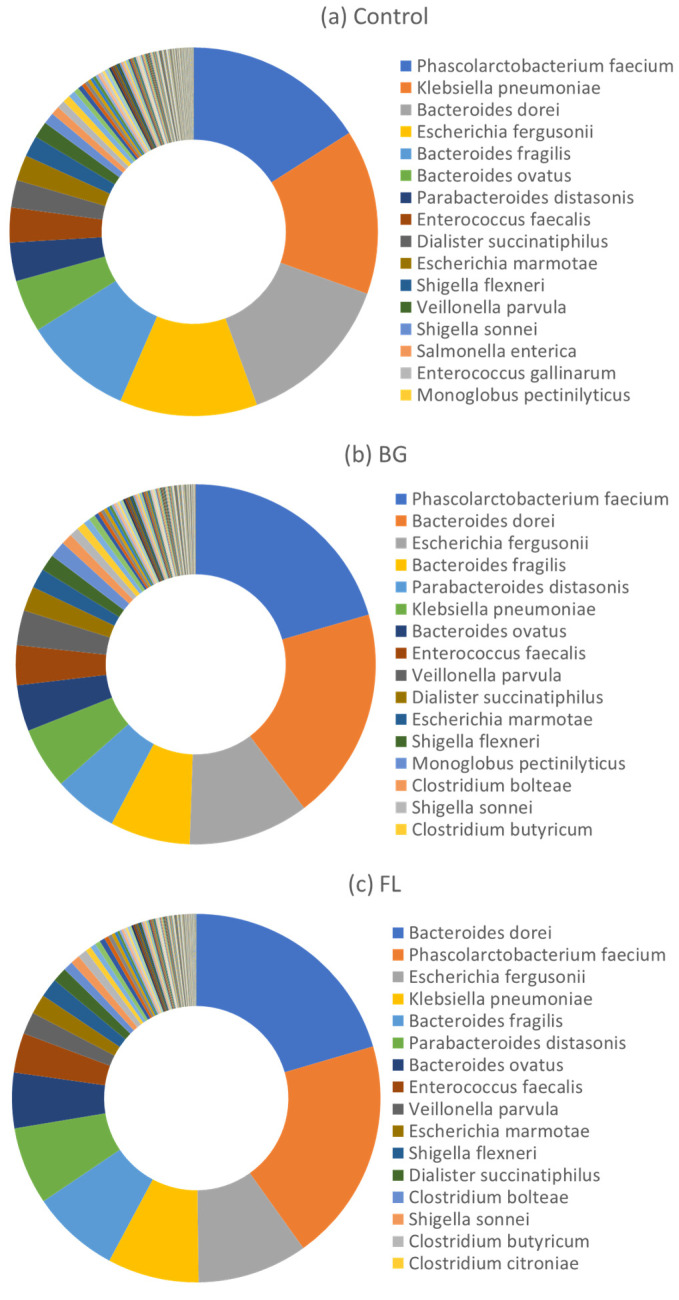
Relative abundance of all species found in (**a**) control, (**b**) BG and (**c**) FL.

**Figure 6:**
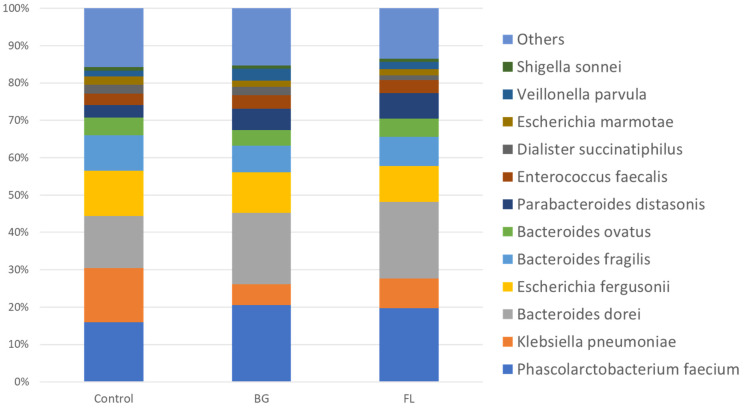
Relative abundance of the top 12 most dominant species.

**Figure 7:**
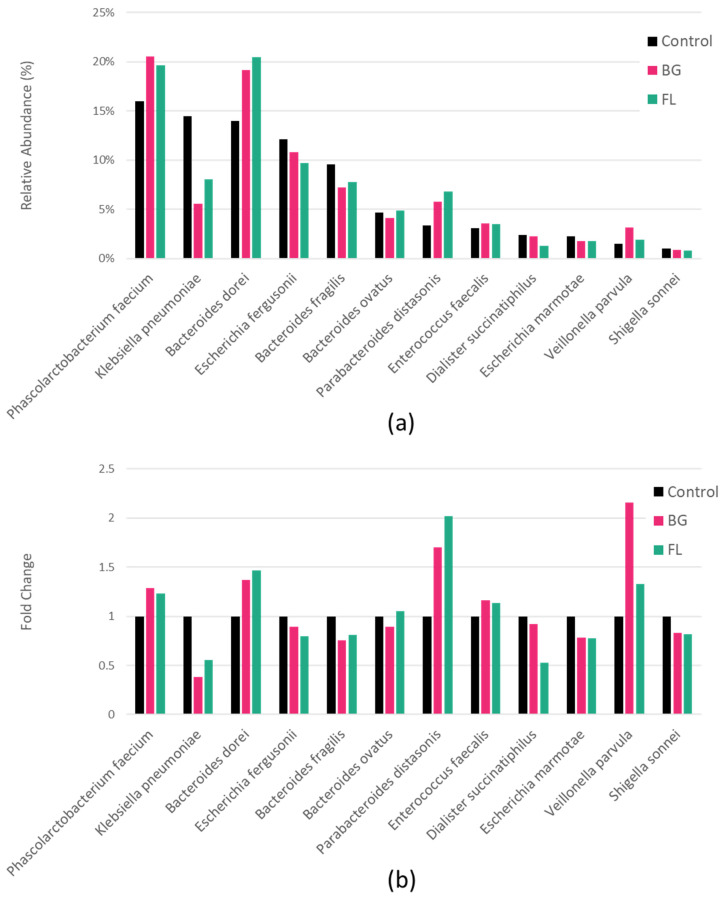
(**a**) Relative abundance of the top 12 most dominant individual species across treatments; (**b**) fold change of the top 12 most dominant species compared to control.

BG and FL had a lower α-diversity than the control in both the Shannon Index and Simpson Index, implying selective enrichments on human fecal microbiota (Figure 4). This was manifested in total microbial compositions in that the top 12 most dominant species found accounted for over 80% of the total community (Figure 5 and Figure 6). Figure 7 depicts the enrichments and/or inhibitions of these 12 species. In short, *Phascolarctobacterium faecium*, *Bacteroides dorei*, *Parabacteroides distasonis* and *Veillonella parvula* were enriched, whereas *Klebsiella pneumoniae*, *Bacteroides fragilis* and *Dialister succinatiphilus* were inhibited.

## 4. Discussion

### 4.1. Fermentability of FL and BG

The complex microbial communities not only colonize our intestinal tract, but also interfere with our immune response, metabolic processes, neurotransmission and health [26]. New terminology such as ‘gut-brain axis’, ‘gut-skin axis’, ‘gut-lung axis’ and more are emerging. It is widely accepted that gut dysbiosis is associated with intestinal and extra-intestinal disease conditions, for example, colitis, diarrhea, allergies, asthma, cirrhosis, metabolic syndrome, hypertension and obesity [27,28,29]. Much research points to interventions targeting the gut microbiota for prevention and reversal of diseased health status or personalized medicine by means of probiotics, prebiotics (the substrate) and post-biotics (the metabolites) and, less commonly, other non-diet factors. The fermentability of polysaccharides is a prerequisite of gut microbiota intervention. In the present study, FL demonstrated an increasing trend in total bacterial count compared to the negative control without a carbon source, indicating that the additional FL was a substrate for certain human fecal microbes which nourished those bacteria and other cross-fed bacteria. Their utilization caused a significant pH drop with a change in the short-chain fatty acid (SCFA) profile and fecal microbial profile. These results proved the presence of microbial fermentation activities of FL. Although BG did not result in obvious differences from the control in terms of bacterial growth, pH and SCFA change, it had a similar impact on microbial composition. FL and BG both reduced the α-diversity of the community. This selective enrichment is a criterion to be defined as a prebiotic as well [30]. Both FL and BG enriched *Phascolactobacterium faecium*, *Bacteroides dorei* and *Parabacteroides distasonis* and inhibited *Klebsiella pneumoniae* and *Bacteroides fragilis*. They are generally beneficial to patients with metabolic and inflammatory disorders like inflammatory bowel disease, arthritis and atherosclerotic lesions. This study demonstrated a good fermentability of FL by human fecal microbiota and a less outstanding fermentability of BG. More investigation on its fermentability by known probiotics, like *Bifidobacterium* and *Lactobacillus,* could be conducted to evaluate their prebiotic potential.

### 4.2. SCFAs Mediate the Interference of Gut Microbiota in Host

The concept of host–gut reciprocal communication has been a hot research interest recently. It was believed that gut microbiota interacts with the host via microbial metabolites. SCFAs played a pivotal role here. They are recognized by G protein-coupled receptors (GPCRs), particularly GPCR41 and GPCR43. These two GPCRs were later renamed as free fatty acid receptor 3 (FFAR3) and FFAR2, respectively. Ligands for them include acetic acid, propionic acid, butyric acid and valeric acid. Various pathways are activated due to different affinities for different SCFA receptors. FL markedly promoted acetic acid production compared to the control. Together with propionic acid, they are of the highest affinity for FFAR2 in humans. FFAR2 is expressed throughout the entire gastrointestinal tract, especially enteroendocrine cells, pancreatic cells and white adipocytes [31]. It is involved in insulin signaling and appetite control via regulating insulin and glucose-stimulated GLP-1 secretion. Acetic acid was found to suppress adipocyte lipolysis and glycerol release in a dose-dependent manner. The intraperitoneal injection of sodium acetate instantly reduced blood fatty acid levels in mice [32]. Lavoie et al. found that FFAR2 is required in maintaining mucosal barrier integrity, the loss of which promotes colon tumorigenesis and death in mice due to increased tumor bacterial load, exhaustion of CD8^+^ T cells and overactivation of dendritic cells [33]. FFAR2-knockout mice exhibited aggravation in colitis, arthritis, asthma and glucose tolerance [31,32,33]. Another study found that long-term acetate deficiency reduced the production of synaptophysin (SYP) in the hippocampus, which impaired cognitive performances in a type 1 diabetes mellitus mice model. SYP secretion was restored with supplementation of exogenous acetate or fecal microbiota transplant via the ‘gut-brain’ axis [34]. Additionally, DSS-colitis mice treated with acetate via enema administration showed faster epithelial wound healing via the activation of c-Jun N-terminal kinase and Rho signaling pathways [35]. FL with an acetic acid-promoting property is a potential medicinal food for colonic and metabolic disorders.

The other two health-promoting members did not resemble acetic acid. Propionic acid production in FL was slightly reduced, whereas butyric acid production was similar. Propionic acid receives attention for its regulating effects on lipogenesis, satiety, and the anti-proliferation of colon cancer cell lines [36]. Many of them overlap with acetic acid. This may be attributed to their common activation of FFAR2 and downstream pathways. Butyric acid is often discussed as energy fuel for colonocytes via β-oxidation because 95% of microbial butyrate is consumed in the colon. Very few, if any, can enter circulation to peripheral tissues [37]. Morrison and Preston described a similar concentration gradient of SCFAs. The main SCFAs flowing in the bloodstream are acetic acid and, to a much lesser extent, propionic acid after local consumption in the colon, leaving acetic acid the highest exposure to peripheral tissues [38]. FL may not exhibit its impact through the energy supply for colonocyte. There was no net change of valeric acid content in FL, while the medium negative control contributed to its increment, implying that the valeric acid generated from the medium was used up in the FL group but not the BG group. Cross-feeding probably took place in the FL group, where some valeric acid-utilizing microbes were supported after a cascade or network of enrichment.

### 4.3. FL and BG Modulate Gut Microbial Profile and Potential Impacts

The impact of food substrates on gut microbiota, especially its composition, is extremely complex because microbial cross-feedings and cascade effects often take place. Polysaccharide digestion usually starts by carbohydrate-active enzymes, such as glycoside hydrolases and polysaccharide lyases, on the cell surface. *Bacteroides spp.* have been reported as primary glycan degraders in complex carbohydrate metabolism on their cell surface. Degraded oligosaccharides are then internalized for further digestion or act as substrates for other microbes [39]. As a member of *Bacteroides*, *Bacteroides dorei* was enriched by BG and FL by nearly 40%. *B. dorei* oral administration with *B. vulgatus* resulted in attenuated atherosclerotic lesion, endotoxemia, lipopolysaccharide production in gut microbes and pro-inflammatory immune responses [40].

There was a noticeable increment of *Parabacteroides distasonis* by over 1.5 to 2-fold. It could transform the bile acid profile by increasing lithocholic acid and ursodeoxycholic acid. The mixture of these two secondary bile acids inhibited hyperlipidemia and repaired gut barrier integrity in obese mice [41]. The oral treatment of *P. distasonis* membrane antigens alleviated acute and chronic intestinal inflammation induced by dextran sulphate sodium, a well-established inflammatory bowel disease inducer in mouse models, via specific antibody responses and the stabilization of intestinal microbiota ecology [42]. Additionally, it produces succinate, contributing to the cross-feeding of other microbes, including *Phascolarctobacterium faecium* [41].

The abundance of *P. faecium* was the highest and increased under both BG and FL supplementation. The acetic acid-generating property of FL may be attributed to this succinate-utilizing bacterium because it is a known substantial acetate or propionate producer. It feeds on succinate produced by other microbes, such as *Bacteroides* and *Parabacteroides* mentioned earlier [43]. It was found in a higher abundance in the healthy one among a pair of healthy and allergic twins [44]. Although *P. faecium* was elevated in rats fed a high-fat diet, its deficit was found to be associated with plenty of health disorders. Digestive and metabolic diseases-susceptible groups (age under 1 and over 60) were linked to a reduced abundance of *P. faecium* [43]. Four gut microbes from *Phascolarctobacterium* and *Bacteriodes*, including *P. faecium*, were significantly reduced in type 2 diabetes mellitus patients [45]. A complete genome of a *P. faceium* strain isolated from a healthy lean donor released in 2021 revealed a complete pathway for vitamin B_12_ biosynthesis [46]. Symptoms of vitamin B_12_ deficiency include fatigue, headaches, palpitations, pale skin, etc. Traditional Chinese Medicine practitioners may treat these symptoms with FL, a medicinal food described as ‘spleen-invigorating’ in TCM documentation [47].

The inhibition of about 50% of *Klebsiella pneumoniae* is another piece of evidence supporting the consumption of BG and FL. *K. pneumoniae* is a notorious, opportunistic nosocomial pathogen. This antibiotic resistance gene (ARG) reservoir accumulates ARGs via mutations and the acquisition of transferable plasmids. It imposes a worldwide public health threat due to its surging infection rate and high mortality [48]. BG and FL slightly reduced the number of *Bacteroides fragilis*, a gut commensal and an opportunistic pathogen [39]. Two other bacteria with ≤3% relative abundance were differentially regulated with mixed effects. *Veillonella parvula* was enriched over two-fold by BG but much less by FL. It is a normal microbe in the mouth, gastrointestinal tract and vagina in humans. Very rarely, it causes infections in the sinuses, lungs, heart, bones and nervous system. Moreover, it can conduct nitrate respiration anaerobically, leading to an increase in IBD patients [49,50]. *Dialister succinatiphilus* was highly inhibited by FL, but mildly by BG. Succinate-decarboxylating *D. succinatiphilus* was positively correlated with liver cirrhosis and negatively associated with Hepatitis B virus infection [51]. This in vitro fermentation had shown impressive microbiota-modulating capabilities of BG and FL. However, it should be emphasized that these SCFA-promoting and microbiota-modulating effects may not resemble the situation of consumption by live animals or humans due to the distinct micro-environment in the gut, the existence of less cultivable bacteria species and the selectivity of fermentation medium. Further studies on BG and FL consumption on the above associated disease conditions could be conducted to validate the effects in vivo.

## 5. Conclusions

Fu Ling, containing over 70% of β-(1 → 3), (1 → 6)-glucans with 93% glucose monomer and a low degree of branching of 0.24, showed good fermentability. It resulted in an increasing trend in bacterial growth, a lowered pH value, and doubled acetic acid production in this in vitro fermentation. Elevated acetate is involved in energy homeostasis, mucosal barrier integrity and cognitive function via FFAR2 activation. While FL was better fermented than its β-glucan, they had similar modulation on microbial composition. They enriched *Phascolarctobacterium faecium*, *Bacteroides dorei* and *Parabacteroides distasonis,* which are shown to possess anti-inflammatory effects in atherosclerosis and colitis and ameliorating effects in type 2 diabetes mellitus, food allergies and obesity. Furthermore, they inhibited opportunistic pathogens *Klebsiella pneumoniae* and *Bacteroides fragilis.* In summary, this study demonstrated a good fermentability of FL by human fecal microbiota, while its polysaccharide extract did not exhibit a better performance. Prior extraction and chemical modification were not necessary for microbial utilization, and the subsequent beneficial impacts brought by gut microbiota composition modulation and microbial metabolites.

## Figures and Tables

**Table 1:** Primers and corresponding sequences employed in fungi identification and authentication.

Primer	Primer Sequence (5′→ 3′)
ITS 1	TCCGTAGGTGAACCTGCGG
ITS 4	TCCTCCGCTTATTGATATGC

**Table 2:** Tailed primers and corresponding sequences used in 16S amplification.

Primer	Primer Sequence (5′ → 3′)
Tailed 27 F ^1^	TTTCTGTTGGTGCTGATATTGCAGAGTTTGATCCTGGCTCAG
Tailed 1492 R ^1^	ACTTGCCTGTCGCTCTATCTTCTACGGYTACCTTGTTACGACTT

^1^ Tailed sequence contained adaptor to Oxford Nanopore Technologies Sequencing platform.

**Table 3:** Percentage monosaccharide composition of FL and FL β-glucan.

Monosaccharide	FL	FL β-Glucan
Arabinose	5.39	n.d. ^1^
Mannose	1.57	n.d.
Glucose	93.04	100.00

^1^ n.d. represents not detected.

**Table 4:** Percentage total and β-glucan content (*w/w*) of FL and its polysaccharide β-glucan.

	FL	FL β-Glucan
Total glucan	76.47 ± 9.61	73.10 ± 6.66 ^n.s.^
Percentage β-(1 → 3), (1 → 6)-glucans	99.31 ± 0.07	99.94 ± 0.02 ^n.s.^

Results are expressed by mean ± SD, analyzed using two-tailed Student *t*-test. n.s., not significant, compared to FL.

**Table 5:** Peak area and molar ratio of glycosidic linkage in FL and FL β-glucan.

Retention Time (min)	Methylated Sugars	Linkage Pattern	FL		FL β-Glucan	
			Peak Area (%)	Molar Ratio	Peak Area (%)	Molar Ratio
17.7	2,3,4,6-Me4-Glc	T-Glc*p* ^1^	10.06	1.00	3.08	1.00
20.7	2,4,6-Me3-Glc	1,3-Glc*p*	76.08	6.96	91.22	27.29
24.6	2,4-Me2-Glc	1,3,6-Glc*p*	13.86	1.17	5.70	1.58
DB ^2^				0.24		0.09

^1^ Glc*p* represents glucopyranose. ^2^ DB = (N_T_ + N_B_)/(N_T_ + N_B_ + N_L_), the degree of branching, where N_T_, N_B_ and N_L_ are numbers of terminal residues, branch residues and linear residues, respectively.

## Data Availability

The data presented in this study can be made available by the corresponding author upon request.
